# The interaction of selenoprotein F (SELENOF) with retinol dehydrogenase 11 (RDH11) implied a role of SELENOF in vitamin A metabolism

**DOI:** 10.1186/s12986-017-0235-x

**Published:** 2018-01-22

**Authors:** Jing Tian, Jiapan Liu, Jieqiong Li, Jingxin Zheng, Lifang Chen, Yujuan Wang, Qiong Liu, Jiazuan Ni

**Affiliations:** 1 College of Life Sciences and Oceanography, Shenzhen Key Laboratory of Marine Bioresources and Eco-environmental Science, Shenzhen University, Shenzhen, 518060 China; 2 College of Life Sciences and Oceanography, Shenzhen Key Laboratory of Microbial Genetic Engineering, Shenzhen University, Shenzhen, 518060 China; 3 College of Life Sciences and Oceanography, Shenzhen Engineering Laboratory for Marine Algal Biotechnology, Shenzhen University, Shenzhen, 518060 China; 4 Department of Neurology, Shenzhen University 1st Affiliated Hospital, Shenzhen Second People’s Hospital, Sungang West Road, Shenzhen, China

**Keywords:** SELENOF (Seleonoprotein F), Yeast two hybrid system, Protein-protein interaction, Retinol dehydrogenase 11 (RDH11), Fluorescence resonance energy transfer (FRET), Co-immunoprecipitation (co-IP), Pull-down, Retinol (vitamin a), Retinaldehyde

## Abstract

**Background:**

Selenoprotein F (SELENOF, was named as 15-kDa selenoprotein) has been reported to play important roles in oxidative stress, endoplasmic reticulum (ER) stress and carcinogenesis. However, the biological function of SELENOF is still unclear.

**Methods:**

A yeast two-hybrid system was used to screen the interactive protein of SELENOF in a human fetal brain cDNA library. The interaction between SELENOF and interactive protein was validated by fluorescence resonance energy transfer (FRET), co-immunoprecipitation (co-IP) and pull-down assays. The production of retinol was detected by high performance liquid chromatograph (HPLC).

**Results:**

Retinol dehydrogenase 11 (RDH11) was found to interact with SELENOF. RDH11 is an enzyme for the reduction of all-trans-retinaldehyde to all-trans-retinol (vitamin A). The production of retinol was decreased by SELENOF overexpression, resulting in more retinaldehyde.

**Conclusions:**

SELENOF interacts with RDH11 and blocks its enzyme activity to reduce all-trans-retinaldehyde.

**Electronic supplementary material:**

The online version of this article (10.1186/s12986-017-0235-x) contains supplementary material, which is available to authorized users.

## Background

Selenium (Se) is a necessary trace element for human health. It primarily exerts its function through selenoproteins in which Se is present in the form of selenocysteine (Sec), which is encoded by UGA, traditionally read as a stop codon in the coding region. SELENOF, initially named as 15-kDa selenoprotein (Sep15) is one of the 25 genes in humans encoding for selenoproteins [1]. It is conserved from worms to human beings, whereas the genes in *C. elegans, B. malayiand Drosophila* encode homologous proteins that contain cysteine in place of selenocysteine [2]. The NMR structure of fruit fly SELENOF shows that the protein contains a thioredoxin-like motif. The redox potential of this motif is between that of thioredoxin and protein disulfideisomerase, so SELENOF was assumed to be a thiol-disulfide oxido-reductase and/or isomerase [3]. The N-terminal signal peptide of SELENOF guides it to localize to the ER [4]. Mouse SELENOF was shown to form a 1:1 complex with UDP-glucose: glycoprotein glucosyltransferase 1 (UGGT1), which is a chaperone involved in the quality control of N-glycosylated proteins in ER mediated by the cysteine-rich domain of SELENOF. Thus, SELENOF could possibly be involved in glycoprotein folding control in ER [5, 6]. Tunicamycin (an N-linked glycosylation inhibitor and is commonly used to induce ER stress experimentally) upregulates the SELENOF protein level, whereas dithiothreitol (DTT, an ER stressor which blocks formation of disulfide bonds in the ER preventing xidative folding of membrane and secretory proteins) promotes SELENOF degradation through the proteasome pathway [7]. SELENOF knock out (KO) mice demonstrated no obvious phenotypes, except the development of nuclear cataracts resulting from improper protein folding and posttranslational modifications that always induce ER stress [8]. The deficiency of SELENOF in Chang liver cells induces ER stress and inhibits cell proliferation and invasion [9]. This outcome is consistent with the results of a knockdown of SELENOF mRNA in a colon cancer cell line, which resulted in the inhibition of tumor cell growth, and lung metastasis [10, 11]. Knockout of SELENOF in mice also prevented the formation of chemically induced aberrant crypts [12]. However, deletion of SELENOF in NIH3T3 cells led to cytoskeleton remodeling and membrane blebbing but not to apoptosis [13]. No ER stress was detected in the liver of SELENOF KO mice either [8]. The data imply that SELENOF plays multiple roles in different tissues or cells. SELENOF is discovered to distribute in brain with high level. It is explored from Atlas dataset that SELENOF gene expression is rich in neurons in olfactory bulb, hippocampus, cerebral cortex, and cerebellar cortex [14, 15]. It also has high transcript levels in mouse neuronal cells of the hippocampus and Purkinje cells of the cerebellar cortex, with expression profiles that are similar to the ER chaperones calnexin (CNX) and oxido-reductase ERp57, which are the major components of the quality control mechanism in ER [7], so SELENOF might play some roles in central neural system (CNS).

Human retinol dehydrogenase 11 RDH11 belongs to the short-chain dehydrogenases/ reductases (SDR) family [16]. It is able to reduce both all-trans- and cis-retinaldehydes into all- trans- and cis- retinol (Vitamin A) [17, 18]. An RDH11 knockout in mice did not affect the retinoid profiles when the mice adapted to dark conditions [17], but the deleterious mutations in the RDH11 gene caused a new syndrome with retinitis pigmentosa [19], which is an inherited degenerative disease in the retina that causes severe vision impairment. Therefore, RDH11 may be related to not only the vitamin A cycle but also retina protection.To obtain insight into the function of SELENOF in the brain, an interactive protein of SELENOF was screened out to be RDH11 with a yeast two-hybrid system and validated with fluorescence resonance energy transfer (FRET), co-immunoprecipitation (co-IP) and pull-down assays in this study. Overexpression of SELENOF in HEK293T cells inhibited enzyme activity of RDH11, implying the function of SELENOF in retinol metabolism.

## Methods

### Amplification and mutagenesis of cDNA encoding SELENO F and plasmid construction

The cDNA encoding human SELENOF was cloned from a human fetal brain cDNA library (Clontech, U.S.A). The primers, restriction enzymes and plasmids constructed for this study are all presented in Table 1. Due to the absence of selenoprotein synthesis machinery in yeast cells, the Sec-coding TGA codons in SELENOF was changed into Cys-coding TGC by using site-directed mutagenesis [20] to generate the SELENOF’ gene, which was then inserted into vector NpGBKT7 (Clontech, U.S.A), pEYFP-N1 (kindly provided by professor Deming Gou from Shenzhen University) and PET28b (Novagen), to generate NpGBKT7-Seleno F′, pECFP-C1-SELENOF’, and SELENOF’-PET28b for Yeast two-hybrid, FRET and pull down experiments, respectively. The plasmids were confirmed to contain the target gene fragments by restriction enzyme analysis and DNA sequencing.

**Table 1:** Primers used for plasmids construction

Plasmids constructed		Sequence
SEL F′-YFP-N1	F	5’-ATGGTAGCGATGGCGGCTGGGC-3′
	R	5’-TTATATGCGTTCCAACTTTTCAC-3′
NpGBKT7-Sel F′	F	5’-TTTGGGGCAGAGTTTTCATCGGAG-3’
	R	5’-TTATATGCGTTCCAACTTTTCAC-3’
RDH11-YFP-N1	F	5’-CGGAATTCATGGTTGAGCTCATGTTCCCGCTGT-3’
	R	5’-TAGGTACCCTGTCTATTGGGAGGCCCAGCAGGTC-3’
RDH11-CFP-C1	F	5’-CGGAATTCCGTTGAGCTCATGTTCCCGCTGT-3’
	R	5’-TAGGTACCTGGGTCCAACTGGCACTGCCTGTTA-3’
SEPT3-CFP-C1	F	5’-ATAAGCTTATTCCAAAGGGCTCCCAGAGAC-3’
	R	5’-TAGGTACCTCATGGGTTACTGTCGTGGCTTT-3’
TUBB4-CFP-C1	F	5’-TAGAATTCACGGGAGATCGTGCACCTGCAGG-3’
	R	5’-TAGGTACCCTAGGCCACCTCCTCCTCCGCCT-3’

### Library screening through yeast two-hybridization

Plasmid NpGBKT7-SELENOF’ was used to screen the human fetal brain cDNA library via the yeast two-hybrid system. Yeast transformation and library screening were performed following the procedures described in the user manuals for the assays (YeastmakerTM Yeast Transformation System 2 and Matchmaker™ Gold Yeast Two-hybrid System, Clontech, U.S.A). Positive yeast colonies from library screening were selected from the quadruple dropout solid medium SD/−Ade/-His/−Leu/−Trp containing X-α-Gal and Aba. Plasmids extracted from the yeast cells were transformed into *E. coli* Top10 competent cells. Prey plasmids were amplified in LB medium containing ampicillin and extracted from *E. coli* cells. The obtained prey plasmids and the NpGBKT7-SELENOF’ bait plasmid were co-transformed into Y2HGold yeast cells and grown on SD /−Leu /−Trp /x-α-gal /Aba solid medium to determine whether the colonies turned blue. The positive prey plasmids were then sequenced and analyzed with the basic local alignment search tool (BLAST).

### Verification of the interaction between SELENOF’ and each prey by fluorescence resonance energy transfer (FRET)

All the procedures were referred to the papers published previously [21, 22]. To verify the interaction between SELENOF’ and each prey, the coding sequences of SELENOF’ and each prey were inserted into expression vectors (pEYFP-N1 and pECFP-C1, a kindly gift from Deming Gou in Shenzhen University) containing enhanced yellow fluorescence protein (YFP) and cyan fluorescence protein (CFP), respectively. For the FRET acceptor photobleaching assay, each pECFP-C1-prey plasmid and pEYFP-N1-SELENOF’ plasmid was co-transfected into Human embryonic kidney HEK293T cells. The transfection procedure was performed according to the lipofectamine 2000 transfection protocol (Thermo Fisher Scientific, USA), and imaging was performed using a confocal laser scanning microscope (Olympus Fluoview FV1000, Tokyo, Japan) with a 60× oil immersion objective and the acceptor photobleaching module. The acceptor signal (yellow fluorescence) was bleached in defined regions of interest (ROI) with 515 nm light at 98% laser power for 60 s. The changes in the donor signal (cyan fluorescence) induced by acceptor photobleaching were quantified by comparing the pre- and post-bleaching images obtained by excitation at 405 nm.The acquired data were also analyzed using the Olympus Fluoview FV1000 Toolbox software. FRET efficiency was calculated as Ipre-bleaching/Ipost-bleaching, where pre-bleaching is the intensity of cyan fluorescence before bleaching in defined ROIs. Distance (r) was calculated as R0 (1/E-1)1/6, where R0 is the determined by outlining an ROI in a region containing no cells. This background value was subtracted from each value obtained in the cells. The mean fluorescence intensity obtained in at least 20 ROIs from at least three different transfected cells was measured for each protein pair. As controls, cells co-transfected with pEYFP-N1-SELENOF’ and pECFP-C1 or pEYFP-N1 and ECFP-C1-prey were also studied.

### Co-IP detection for protein-protein interaction

Approximately 24 μg of the SELENOF-HA-SelExpress1 plasmid and RDH11-pEYFP-N1 was co-transfected into 1 × 10^6^HEK293T cells seeded in 100 mm culture dishes (pSelExpress1 is a plasmid expressing recombinant selenoprotein with high efficiency and was a kind gift from Prof. Gladyshev at the Harvard Medical School). pEYFP-N1, SELENOF-HA-SelExpress1, RDH11-pEYFP-N1 and HA-SelExpress1 were transfected into cells as the control conditions. Forty eight hours after transfection, the cells were lysed in RIPA solution (Bonataike, Shenzhen, China). The lysates were centrifuged at 12,000 g for 30 min at 4 °C. The supernatants were collected, and the total protein amount was determined using the bicinchoninic acid (BCA) protein assay kit (Beyotime, China). One mg of supernatant protein from each group was mixed with protein G plus-agarose beads at 4 °C for 1 h to preclear the sample for minimizing nonspecific binding of proteins. Then, the precleared supernatant was incubated with 2 μg of anti-HA or anti-GFP antibody for 1 h at 4 °C, followed by incubation with protein G-agarose beads overnight at 4 °C. The agarose beads were spun down at 2000 g for 2 min and washed three times with RIPA lysis buffer and twice with PBS.

The collected beads were boiled in 5× loading buffer, and the supernatant was separated by SDS-PAGE and analyzed by Western blotting (WB) using the primary antibody for anti-GFP or the anti-HA antibody.

### His pull-down assay

SELENOF’-PET28b was transformed into *E.coli BL 21 (DE3)* and recombinant SELENOF’ was induced by 0.4 mM IPTG at 30 degree for about 16 h. Cell lysates of *E. coli* containing recombinant his-tagged SELENOF’ were immobilized on Ni-NTA beads, and the purified protein was incubated on the beads with RDH11-YFP, which was overexpressed in HEK293T cells. The beads containing the protein interaction complex were washed four times with lysis buffer, boiled in 5× loading buffer and analyzed by WB using an anti-GFP monoclonal antibody.

### Analysis of RDH11 enzymatic activity affected by SELENOF

Catalytic activity of RDH11 was assayed in 25 mmol/L phosphate buffer saline (PBS), at 37 °C (reaction buffer) in siliconized glass tubes. The reductive activity of RDH11 toward all- trans-retinaldehyde substrate was analyzed using all- trans-retinol and all- trans-retinaldehyde (Roch). The stock solution of all- trans-retinaldehyde substrate was dissolved in DMSO. The 500 μl reactions were started by the addition of cofactor 0.3 mmol/L NADPH-Na4 and carried out for 15 min at 37 °C with 200 rpm shaking. The amount of cell lysates in the reaction mixture is around 250 μg. The reactions were terminated by the addition of an equal volume of cold methanol: n-butyl alcohol 95:5 supplemented with 100 μg/ml butylated hydroxytoluene. Retinoids were analyzed using a Waters Alliance HPLC system. Elution was monitored at 325 nm. Ten-microliter aliquots were analyzed by reverse-phase HPLC (SHIMADZU, Japan) using a C18 column (4.6 × 150 mm), and the mobile phase consisted of acetonitrile: 30 mmol/L ammonium acetate (85, 15, *v*/v). The flow rate was 1.5 ml/min. Under these conditions, all- trans-retinol and all-trans-retinaldehyde eluted at around 11.27 and 12.64 min, respectively. The reaction products were quantified by summing the areas of both peaks of retinol and retinal. The ratios of the produced retinol were analyzed.

### Statistical analysis

Results are presented as mean ± SEM. Statistical analysiswas performed by ANOVA and student t-test (Graphpad software, San Diego, CA, USA). A value of *P* < 0.05 was considered as statistically significant.

## Results

### The interactive protein of SELENOF’ screened with a yeast two-hybrid system from a human fetal-brain cDNA library

Before screening, NpGBKT7-SELENOF’ was transformed into Y2H Gold yeast cells, and no self-activation was observed. The NpGBKT7-SELENOF’ containing yeast colonies were then used to screen the fetal brain cDNA library. On the corresponding selection plates (SD/−Ade/-His/−Leu/−Trp) containing X-α-Gal and aureobasidin A (Aba), several blue (positive) colonies were grown (Additional file 1: Figure S1). The plasmids from these colonies were extracted and transformed into *E. coli* Top10 competent cells separately. The bait plasmid and each screened prey plasmid were then re-transformed into Y2H Gold yeast cells, followed by plate selection (SD/−Trp/−Leu/-His/−Ade/X-α-gal/Aba). The prey plasmids in three positive colonies following re-transformation were subjected to sequencing and bioinformatics analysis with the Basic Local Alignment Search Tool (BLAST). They were found to be *Homo sapiens* retinol dehydrogenase 11 (RDH11), *Homo sapiens* tubulin, beta 4 (TUBB4) and *Homo sapiens* Septin 3 (SEPT3).

### FRET verification of protein-protein interaction

**Fig. 1:**
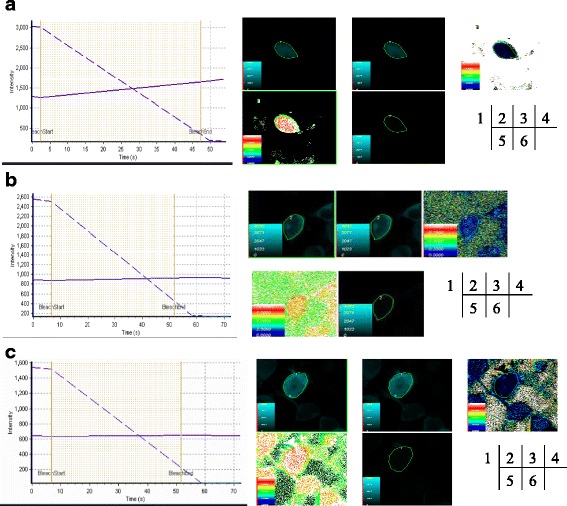
Detection of protein-protein interaction by acceptor bleaching method. HEK293T cells were co-transfected with Sel F’-pEYFP-N1 and pECFP-C1-RDH11 for sample tests (**a**). Then, cells were co-transfected with pEYFP-N1 and pECFP-C1-RDH11 (**b**) with ECFP-C1 and SEL F’-pEYFP-N1 (**c**) as negative controls. (1) Photobleaching curves (solid lines for donor fluorescence and dashed lines for receptor fluorescence). The region of interest (ROI) was bleached at 515 nm. (2) The fluorescence images of donors before bleaching. (3) The fluorescence images of donors after bleaching. (4) Donor fluorescence increments before and after bleaching. (5) Diagram of the distance between donor and receptor. (6) FRET efficiency diagram

To verify the interaction between SELENOF’ and the prey plasmids, FRET analysis of the acceptor photo bleaching was performed. The results showed that the estimated distance between the SELENOF’-YFP donor and the CFP-RDH11 acceptor was 5.51 nm ± 0.235 nm (*n* = 8) (Fig. 1a). The energy transfer efficiency between the proteins was calculated to be 29.21% ± 6.1% (*n* = 8) (Fig. 1a). The FRET efficiency of control cells co-transfected with pEYFP-N1 and ECFP-C1-RDH11 was calculated to be 6.16% ± 1.7% (*n* = 8) (Fig. 1b), and the distance was estimated to be 8.51 nm ± 0.361 nm (*n* = 8, Fig. 1b). The FRET efficiency of control cells co-transfected with pEYFP-N1-SELENOF’ and pECFP-C1 was calculated to be 5.28% ± 2.3% (*n* = 8) (Fig. 1c), and the distance was estimated to be 9.12 nm ± 0.251 nm (*n* = 8, Fig. 1c).

The results showed that the fluorescence of the CFP-RDH11 donor significantly increased after the receptor was bleached (Fig. 1a), but this was not observed in control cells (Figure 1b and c). The distance between the CFP-RDH11 donor and the SELENOF’-YFP receptor was less than 10 nm, so the interaction between SELENOF’ and RDH11 was confirmed. FRET assays were also used to detect interactions between SELENOF’ and TUBB4 or SEPT3, but no significant FRET efficiency was detected (Data not shown). Therefore, the following experiments were used to verify the interaction between RDH11 and SELENOF’. RDH 11, TUBB4 and SEPT3 were screened as SelenoF’ interaction proteins in human embryo brain cDNA library using yeast two-hybrid system. All the interactions between preys and SELENOF’ were tested by FRET assay. Only RDH11 and SELENOF’ showed higher interaction efficiency. The interaction between RDH11 and SELENOF’ was further verified by Co-IP and pull down assay.

### Co-IP verification of protein-protein interactions

**Fig. 2:**
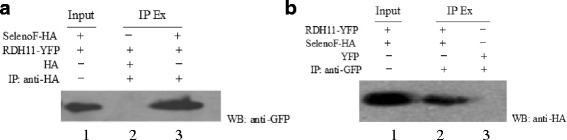
Verification of the interaction between SELENOF and RDH11 by Co-Immunoprecipitation (Co-IP) assay. HEK293T cells were co-transfected with plasmids RDH11-YFP-N1 and SelenoF-HA-SelExpress1 (or RDH11-YFP-N1 and HA-SelExpress1/SELENOF-HA-SelExpress1 and YFP-N1 as the negative controls) . The supernatants of the cell lysates were immunoprecipitated with an anti-HA antibody (**a**) or anti-GFP antibody (**b**) and analyzed by western blot (WB) using an anti-GFP antibody (**a**) or anti-HA antibody (**b**). The supernatant of the cell lysate, named Input, was used as a positive control for WB analysis and probed with anti-HA (**b**) or anti-GFP (**a**) antibody. IP Ex: IP extract

To further confirm the interaction between SELENOF and RDH11, co-IP assays were performed. The constructed plasmids SELENOF-HA-SelExpress1 and RDH11-pEYFP were co-transfected into HEK293T cells. An antibody against HA was used to detect IP HA-tagged SELENOF from cell extracts. The isolated proteins were analyzed by WB using an anti-GFP antibody to detect YFP-tagged RDH11 (YFP contains Tyr203 instead of Thr203 in GFP, so it is able to be detected by anti-GFP antibody). A specific association between HA-tagged SELENOF and GFP-tagged RDH11 is shown in lane 2 of Fig. 2a. Conversely, a GFP antibody was used to identify IP EYFP-tagged RDH11, and an HA antibody was used to probe for HA-tagged SELENOF. The association of SELENOF with RDH11 is also shown in lane 2 of Fig. 2B. A specific association between HA-tagged SELENOF and RDH11 is shown in Fig. 2, which further confirms the interaction between SELENOF and RDH11 in mammalian cells.

### Pull-down verification of the protein-protein interaction

**Fig. 3:**
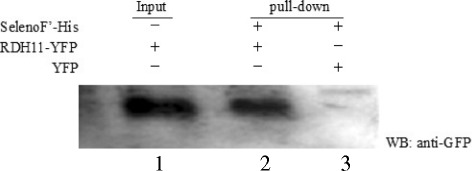
Verification of the interaction between SELENOF’ and RDH11 by pull-downassay. SELENOF’-PET28b was transfermated into *E.coli BL21 (DE3)* and recombinant SELENOF’-his was purified by Ni-NTA; RDH11-EYFP-N1 was transfected into HEK293T celss. Cell lysates containing RDH11-EYFP-N1 and purified SelenoF’-His were incubated with Ni-NTA resin (pull-down) and then detected by anti-GFP antibody. The supernatant of the cell lysate containing RDH11-EYFP-N1, named Input, was used as a positive control for WB analysis probed with an anti-GFP antibody. Cell lysates containing the EYFP-N1 vector and purified SelenoF’-His were used as negative controls

A pull-down assay was performed to further study the interaction between SELENOF’ and RDH11 in mammalian cells. The recombinant expression of the SELENOF’-His fused protein was purified with pNi-NTA (affinity chromatography) (lane 5 of Fig. 3). RDH11-EYFP-N1 plasmids were transfected into HEK293T cells, and the empty vector pEYFP-N1 was used as the negative control.

An antibody against His was used to separate IP EYFP-tagged RDH11 from cell extracts. The isolated proteins were analyzed by WB using an anti-GFP antibody. A specific association between SELENOF’-His and RDH11-EYFP is shown in lane 2 of Fig. 3, which further confirmed the interaction between SELENOF’ and RDH11.

### Reductase activity of RDH11 affected by SELENOF

**Fig. 4:**
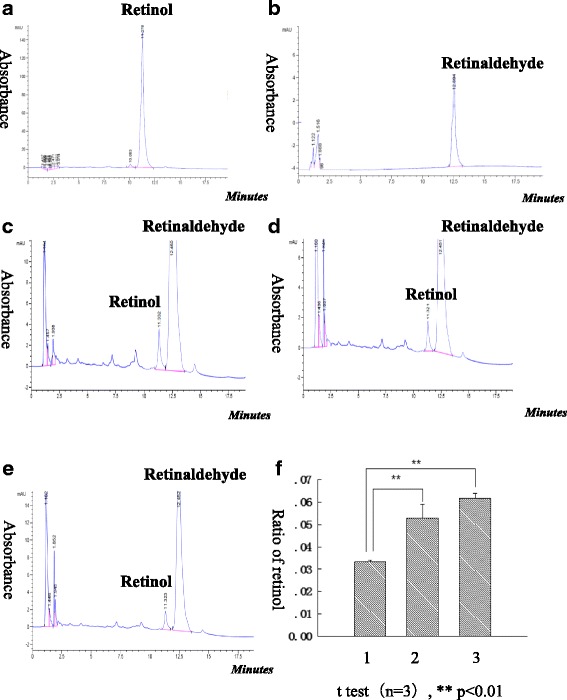
The reductase activity of RDH11 toward all-trans-retinaldehyde affected by SELENOF. **a**.All-trans-retinol standard, it was eluted at 11.27 min; **b**. all-trans-retinal standard, it was eluted at 12.64 min; **c**. Homogenates (250 μl (concertiaon: 1 μg/μl) per reaction) of HEK293 T cells transfected with RDH11-YFP-N1 and HA-SelExpress1 blank vector; **d**. Homogenates (250 μl per reaction) of HEK293 T cells transfected with YFP-N1 blank vector and HA-SelenoF-SelExpress1; **e**. Homogenates (250 μl per reaction) of HEK293 T cells transfected with RDH11-YFP-N1 and HA-SELENOF-SelExpress1; **f**. Data analysis of all-trans-retinol produced in above **c**, **d**, **e** groups. The number 1, 2, 3 were corresponding to the group **e**, **d**, **c** respectively. The reaction products were quantified by summing the areas of both peaks of retinol and retinaldehyde. The ratios of the produced retinol were analyzed. The cell homogenates were incubated with 0.3 mmol/L all-trans-retinaldehyde in the presence of 0.3 mmol/L NADPH-Na4 for 15 min at 37 °C with 200 rpm shaking. The reactions were terminated by the addition of an equal volume of cold methanol: n-butyl alcohol 95:5 supplemented with 100 μg/ml butylated hydroxytoluene. Ten-microliter aliquots were analysed by reverse-phase HPLC using a C18 column. Mobile phase was acetonitrile: 30 mmol/L ammonium acetate (85: 15, *v*/v). The flow rate was 1.5 ml/min. Under these conditions, all-trans-retinol and all-trans-retinaldehyde eluted at around 11.27 and 12.64 min, respectively. Elution was monitored theabsorbance at 325 nm

It was reported that RDH11 was most efficient as an all-trans-retinal reductase [18], therefore the catalytic activity of RDH11 affected by its interacting protein SELENOF was detected by addition of substrate all-trans-retinaldehyde and cofactor NADPH. Expression level of recombinant SELENOF-HA and RDH11-YFP were detected by Western Blot (Additional file 2: Figure S2). As analyzed by reverse-phase HPLC, the peaks of all-trans-retinol and all-trans-retinaldehyde corresponding to the standards were detected (Fig. 4c, d & e ). The standard of all-trans-retinol or all-trans-retinaldehyde was analyzed by reverse-phase HPLC and eluted at around 11.3 or 12.4 min (Fig. 4a & b). The product of all-trans-retinaldehyde reduction catalyzed by RDH11 was all-trans-retinol (Fig. 4c, d & e).Therefore, the reaction products were quantified by ratio of both peaks areas of all-trans-retinol and all-trans-retinaldehyde. The ratios of retinol were calculated and analyzed (Fig. 4f). In the reaction containing cell lysates with only overexpressed SELENOF, some all-trans-retinol was produced (Fig. 4f, column 2), suggestingSELENOF may reduce all-trans-retinaldehyde into all-trans-retinol. The reductase activity of recombinant SELENOF’ was also detected in this study (Additional file 3: Figure S3). Surprisingly, in the reaction containing cell lysates with overexpressed RDH11 and SELENOF, the produced all-trans-retinol is lowest (Fig. 4f, column 1). Perhaps the over expressed SELENOF and RDH11 blocked the retinal reductase activity of each other. The results suggested that SELENOF decreased the retinal reductase activity of RDH11 in HEK 293 T cells.

## Discussion

The co-localizations of RDH11 and SELENOF at ER [23, 24] provide the possible interaction between them. The tissue distributions of RDH11 and SELENOF are also identified to be overlapped. Although RDH11 is involved in the retinal pigment epithelium (RPE) during the retinoid visual cycle, it is found in different tissues such as brain, testis and prostate [25]. SELENOF mRNA was also detected to be expressed highly in all above tissues [26]. The overlapped tissue distributions may imply that both of SELENOF and RDH11 are involved in the same biological processes. The results in this study suggested both of SELENOF and RDH11 might reduce all-trans-retinaldehyde into all-trans-retinol, but over expressed SELENOF and RDH11 inhibited the enzyme activity of each other.

RDH11 belongs to the short-chain dehydrogenases/reductases (SDR) family [16] and exhibits more efficient enzyme activity for reduction of all-trans-retinaldehyde than oxidation of all-trans-retinol (vitamin A, VA) [23]. The results of this study verify this, and detect that over expressed SELENOF inhibits the exogenous RDH11 retinal reductase activity, suggesting that SELENOF is possibly involved in VA metabolism. VA is essential in visual cycle, where 11-cis-retinaldehyde derived from VA associates with opsin protein and then it isomerizes to all-trans-retinaldehyde in response to light, activating the opsin and triggering visual neuronal response [27]. After that, all-trans-retinaldehyde is reduced into all-trans-retinol by reductases generally, stored in retinal pigment epithelium (RPE) and then transformed into 11-cis-retinaldehyde involved in a new visual cycle [28]. Retinol dehydrogenases (RDHs) including RDH11, are responsible for oxidizing 11-cis-retinol to 11-cis-retinaldehyde in RPE and reducing all-trans-retinaldehyde to all-trans-retinol in rod photoreceptor, depending on different cofactors [28]. Cofactor NADPH has been used in this study, so the reductive activity of RDH11 is detected, which is consistent with the previous study [29]. VA and its metabolites (retinoids), such as retinoic acid (RA), are essential not only in visual cycle but also in the development, maintenance and morphogenesis of the central nervous system (CNS) [30]. VA deficiency is positively correlated with cognitive decline in the elderly population; prenatal marginal VA deficiency exacerbates learning and memory deficits in AD model mice and VA supplementation attenuates these affects [31]. A mid-life VA supplementation during 4 months prevents spatial memory decline in 17-month-old rats and improves the dendritic arborisation of new born immature neurons by inducing an increase in the intracellular availability of retinoid acid (RA) [32]. RA is required in growth and development, such as neurogenesis, cardiogenesis, body axis extension, and development of the forelimb buds, foregut, and eye [33]. It is produced from VA in the body by two sequential oxidation steps that convert retinol first to an aldehyde (retinaldehyde) and then to a carboxylic acid, RA [34]. Retinol dehydrogenase 10 (RDH10) was reported to control the step retinol to retinaldehyde in embryo development [35]. In our results, RDH11 has been confirmed to reduce retinaldehyde to retinol, SELENOF has also been detected to has the enzyme activity, but SELENOF overexpression inhibits the production of retinol. It seems that the interaction between SELENOF and RDH11 blocks the reaction from retinaldehyde to retinol or just blocks the enzyme active sites of both RDH11 and SELENOF As a result, the aldehyde production has been increased and then might probably convert to VR to perform important roles insome physiological processes. But the mechanism should be studied in the future work.Although no data is published on the function of SELENOF in brain, several papers have been reported that selenium deficiency is associated with cognitive decline [36] as well as chemicals or compounds containing selenium protect Alzheimer’s disease (AD, one of the neurodegenerative diseases) [37–39]. The expression level of SELENOF is regulated by dietary selenium [12], so that selenium supplementary may increase SELENOF expression level. High level of SELENOF in brain might interact with RDH11 and probably promote the production of aldehyde and VR. The study may provide a new clue for the physiological roles of SELENOF. Future experiments in this direction may lead to a better understanding of the association between RDH11, SELENOF and VA metabolism.

## Conclusions

A SELENOF interactive protein, RDH11, was screened out with the yeast two-hybrid system against a human fetal brain cDNA library and verified by FRET, co-IP and a pull-down assay. The production of retinol converted from retinaldehyde was inhibited by SELENOF implied its role, WHICH revealed the possible role of SELENOF in the vitamin A metabolism.

## Additional files

Additional file 1: Figure S1. Yeast two-hybrid screening of the human fetal brain cDNA library using the SELENOF′ gene as a bait. (A) Plasmids carrying the fetal brain cDNA library were co-transformed into the NpGBKT7-SELENOF′-containing yeast cells and screened by the selection plate for blue colonies; Y2H Gold yeast cells in (E)–(F) were co-transformed with plasmids NpGBKT7-SELENOF′ and preys 15–1(D), 15–2(E), 15–3(F); or with plasmids pGBKT7-Lam and pADT7-T as the negative control (C); or with plasmids pGBKT7-p53 and pADT7-T as the positive control (G), followed by selection on SD/−Trp/−Leu/-His/−Ade/X-α-gal/Aba plates. (DOCX 54 kb)

Additional file 2: Figure S2. Expression level of recombinant SELENOF-HA or RDH11-YFP. The overexpressed SELENOF-HA or RDHA11-YFP was detected by anti-HA antibody (A) or anti-GFP antibody (B). In part A, lane 1 shows HEK293T cells co-transfected with SELENOF-HA-SelExpress1 and RDH11-YFP; lane 2 shows HEK293T cells co-transfected with plasmids SELENOF-HA-SelExpress1 and EYFP empty vector. In part B, lane 1 shows HEK293T cells co-transfected with plasmids SELENOF-HA-SelExpress1 and RDH11-YFP; lane 2 shows HEK293T cells co-transfected with plasmids HA-SelExpress1 empty vector and RDH11-YFP. (DOCX 84 kb)

Additional file 3: Figure S3. The reductase activity of SELENOF’ toward all-trans-retinaldehyde. The plasmid SELENOF’-PET28b was transfected into *E*.c*oli* (BL21) and recombinant SelenoF’ was induced and then the cell lysates were used to detect the enzyme activity to reduce all-trans-retinaldehyde (column A); Empty vector PET28b was transfeced into *E.coli* (BL21) and used as the negative control (column B). (DOCX 53 kb)

## Abbreviations

Co-IP: Co-immunoprecipitation
FRET: Fluorescence resonance energy transfer
HPLC: High performance liquid chromatography
PBS: Phosphate buffer saline
RDH11: Retinol dehydrogenase 11
SELENOF: selenoprotein F

## References

[1] Gladyshev VN, Arnér ES, Berry MJ, Brigelius-Flohé R, Bruford EA, Burk RF, Carlson BA, Castellano S, Chavatte L, Conrad M, Copeland PR, Diamond AM, Driscoll DM, Ferreiro A, Flohé L, Green FR, Guigó R, Handy DE, Hatfield DL, Hesketh J, Hoffmann PR, Holmgren A, Hondal RJ, Howard MT, Huang K, Kim HY, Kim IY, Köhrle J, Krol A, Kryukov GV, Lee BJ, Lee BC, Lei XG, Liu Q, Lescure A, Lobanov AV, Loscalzo J, Maiorino M, Mariotti M, Sandeep Prabhu K, Rayman MP, Rozovsky S, Salinas G, Schmidt EE, Schomburg L, Schweizer U, Simonović M, Sunde RA, Tsuji PA, Tweedie S, Ursini F, Whanger PD, Zhang Y (2016). Selenoprotein Gene Nomenclature. J Biol Chem.

[2] Gladyshev VN, Jeang KT, Wootton JC, Hatfield DLA. New human selenium-containing protein. J Biol Chem. 1998;273(15):8910–5. 10.1074/jbc.273.15.8910 9535873

[3] Ferguson AD, Labunskyy VM, Fomenko DE, Arac D, Chelliah Y, Amezcua CA, Rizo J, Gladyshev VN, Deisenhofer JNMR (2006). Structures of the Selenoproteins Sep15 and SelM reveal redox activity of a new Thioredoxin-like family. J Biol Chem.

[4] Labunskyy VM, Hatfield DL, Gladyshev VN (2007). The Sep15 protein family: roles in disulfide bond formation and quality control in the endoplasmic reticulum. IUBMB Life.

[5] Korotkov KV, Kumaraswamy E, Zhou Y, Hatfield DL, Gladyshev VN (2001). Association between the 15-kDa Selenoprotein and UDP-glucose:glycoprotein glucosyltransferase in the endoplasmic reticulum of mammalian cells. J Biol Chem.

[6] Labunskyy VM, Ferguson AD, Fomenko DE, Chelliah Y, Hatfield DL, Gladyshev VN, Novel Cysteine-rich A (2005). Domain of Sep15 mediates the interaction with UDP-glucose:glycoprotein glucosyltransferase. J Biol Chem.

[7] Labunskyy VM, Yoo MH, Hatfield DL, Gladyshev VN (2009). Sep15, a Thioredoxin-like Selenoprotein, is involved in the unfolded protein response and differentially regulated by adaptive and acute ER stresses. Biochemistry.

[8] Kasaikina MV, Fomenko DE, Labunskyy VM, Lachke SA, Qiu WY, Moncaster JA, Zhang J, Wojnarowicz MW, Natarajan SK, Malinouski M, Schweizer U, Tsuji PA, Carlson BA, Maas RL, Lou MF, Goldstein LE, Hatfield DL, Gladyshev VN (2011). Roles of the 15-kDa Selenoprotein (Sep15) in redox homeostasis and cataract development revealed by the analysis of Sep 15 knockout mice. J Biol Chem.

[9] Bang J, Huh JH, Na JW, Lu Q, Carlson BA, Tobe R, Tsuji PA, Gladyshev VN, Hatfield DL, Lee B (2015). Cell proliferation and motility are inhibited by G1 phase arrest in 15-kDa Selenoprotein-deficient Chang liver cells. J. Mol Cells.

[10] Penney KL, Schumacher FR, Li H, Kraft P, Morris JS, Kurth T, Mucci LA, Hunter DJ, Kantoff PW, Stampfer MJ, Ma J (2010). A large prospective study of Sep 15 genetic variation, interaction with plasma selenium levels and prostate cancer risk and survival. Cancer Prev Res.

[11] Tsuji PA, Naranjo-Suarez S, Carlson BA, Tobe R, Yoo MH, Davis CD (2011). Deficiency in the 15 kDa Selenoprotein inhibits human colon cancer cell growth. Nutrients.

[12] Tsuji PA, Carlson BA, Naranjo-Suarez S, Yoo MH, XM X, Fomenko DE, Gladyshev VN, Hatfield DL, Davis CD (2012). Knockout of the 15 kDa Selenoprotein protects against chemically-induced aberrant crypt formation in mice. PLoS One.

[13] Bang J, Huh JH, Na JW, Shim M, Carlson BA, Tobe R, Tsuji PA, Gladyshev VN, Hatfield DL, Lee BJ (2015). Deficiency of the 15-kDa Selenoprotein led to cytoskeleton remodeling and non-apoptotic membrane Blebbing through a RhoA/ROCK pathway. Biochem Biophys Res Commun.

[14] Zhang Y, Zhou Y, Schweizer U, Savaskan NE, Hua D, Kipnis J, Hatfield DL, Gladyshev VN (2008). Comparative analysis of Selenocysteine machinery and Selenoproteome gene expression in mouse brain. J Biol Chem.

[15] Solovyev ND (2015). Importance of selenium and selenoprotein for brain function: from antioxidant protection to neuronal signalling. J Inorg Biochem.

[16] Lhor M, Méthot M, Horchani H, Salesse C (2015). Structure of the N-terminal segment of human retinol dehydrogenase 11 and its preferential lipid binding using model membranes. Biochim Biophys Acta.

[17] Kasus-Jacobi A, Ou J, Birch DG, Locke KG, Shelton JM, Richardson JA, Murphy AJ, Valenzuela DM, Yancopoulos GD, Edwards AO (2005). Functional characterization of mouse RDH11 as a retinol dehydrogenase involved in dark adaptation in vivo. J Biol Chem.

[18] Kiser PD, Golczak M, Maeda A, Palczewski K (2012). Key enzymes of the retinoid (visual) cycle in vertebrate retina. Biochim Biophys Acta.

[19] Xie YA, Lee W, Cai C, Gambin T, Nõupuu K, Sujirakul T, Ayuso C, Jhangiani S, Muzny D, Boerwinkle E, Gibbs R, Greenstein VC, Lupski JR, Tsang SH, Allikmets R (2014). New syndrome with retinitis Pigmentosa is caused by nonsense mutations in retinol dehydrogenase RDH11. Hum Mol Genet.

[20] Tian J, Liu Q, Dong QX, Ni J (2010). A new method for multi-site-directed mutagenesis. Anal Biochem.

[21] Qiao X, Tian J, Chen P, Wang C, Ni J, Liu Q (2013). Galectin-1 is an interactive protein of Selenoprotein M in the brain. Int J Mol Sci.

[22] Du X, Qiu S, Wang Z, Wang R, Wang C, Tian J, Liu Q (2014). Direct interaction between Selenoprotein P and tubulin. Int J Mol Sci.

[23] Kedishvili NY, Chumakova OV, Chetyrkin SV, Belyaeva OV, Lapshina EA, Lin DW, Matsumura M, Nelson P (2002). Evidence that the human gene for prostate short-chain dehydrogenase/reductase (*PSDR1*) encodes a novel retinal reductase (RalR1). J Biol Chem.

[24] Shchedrina VA, Zhang Y, Labunskyy VM, Hatfield DL, Gladyshev VN (2010). Structure-function relations, physiological roles, and evolution of mammalian ER-resident Selenoproteins. Antioxid Redox Signal.

[25] Lin BY, White JT, Ferguson C, Wang SY, Vessella R, Bumgarner R, True LD, Hood L, Nelson PS (2001). Prostate short-chain dehydrogenase reductase 1 (PSDR1): a new member of the short-chain steroid dehydrogenase/reductase family highly expressed in normal and neoplastic prostate epithelium. Cancer Res.

[26] Kumaraswamy E, Malykh A, Korotkov KV, Kozyavkin S, Hu Y, Kwon SY, Moustafa ME, Carlson BA, Berry MJ, Lee BJ, Hatfield DL, Diamond AM, Gladyshev VN (2000). Structure-expression relationships of the 15-kDa selenoprotein gene. Possible role of the protein in cancer etiology. J Biol Chem.

[27] Sonoda T, Lee SKA (2016). Novel role for the visual retinoid cycle in Melanopsin chromophore regeneration. J Neurosci.

[28] Haeseleer F, Jang GF, Imanishi Y, Driessen CA, Matsumura M, Nelson PS, Palczewski K (2002). Dual-substrate specificity short chain retinol dehydrogenases from the vertebrate retina. J Biol Chem.

[29] Cui YH, Jing CX, Pan HW (2013). Association of Blood Antioxidants and Vitamins with risk of age-related cataract: a meta-analysis of observational studies. Am J Clin Nutr.

[30] Maden M (2007). Retinoic acid in the development, regeneration and maintenance of the nervous system. Nat Rev Neurosci.

[31] Zeng J, Chen L, Wang Z, Chen Q, Fan Z, Jiang H, Wu Y, Ren L, Chen J, Li T, Song W (2017). Marginal vitamin a deficiency facilitates Alzheimer's pathogenesis. Acta Neuropathol.

[32] Sahu B, Maeda A. Retinol Dehydrogenases Regulate Vitamin A Metabolism for Visual Function. Nutrients. 2016; 8(11): pii: E746. 10.3390/nu8110746 PMC5133129 27879662

[33] Kam RK, Deng Y, Chen Y, Zhao H (2012). Retinoic acid synthesis and functions in early embryonic development. Cell Biosci.

[34] Duester G (2008). Retinoic acid synthesis and signaling during early organogenesis. Cell.

[35] Sandell LL, Lynn ML, Inman KE, McDowell W, Trainor PA (2012). RDH10 oxidation of vitamin a is a critical control step in synthesis of retinoic acid during mouse embryogenesis. PLoS One.

[36] Pillai R, Uyehara-Lock JH, Bellinger FP (2014). Selenium and selenoprotein function in brain disorders. IUBMB Life.

[37] van Eersel J, Ke YD, Liu X, Delerue F, Kril JJ, Götz J, Ittner LM. Sodium selenate mitigates tau pathology, neurodegeneration, and functional deficits in Alzheimer's disease models. Proc Natl Acad Sci U S A. 2010; 431 107(31):13888–93. 10.1073/pnas.1009038107 PMC2922247 20643941

[38] Zhang ZH, QY W, Zheng R, Chen C, Chen Y, Liu Q, Hoffmann PR, Ni JZ, Song GL (2017). Selenomethionine mitigates cognitive decline by targeting both tau hyperphosphorylation and Autophagic clearance in an Alzheimer's disease mouse model. J Neurosci.

[39] Xie Y, Tan Y, Zheng Y, Du X, Liu Q (2017). Ebselen ameliorates β-amyloid pathology, tau pathology, and cognitive impairment in triple-transgenic Alzheimer's disease mice. J Biol Inorg Chem.

