# Supplementary material for: The interaction of selenoprotein F (SELENOF) with retinol dehydrogenase 11 (RDH11) implied a role of SELENOF in vitamin A metabolism
Source: Nutr Metab (Lond). 2018 Jan 22;15:7. doi: 10.1186/s12986-017-0235-x (PMC5778809; doi:10.1186/s12986-017-0235-x)
Supplement: Supplementary file 1 — Yeast two-hybrid screening of the human fetal brain cDNA library using the SELENOF′ gene as a bait. (A) Plasmids carrying the fetal brain cDNA library were co-transformed into the NpGBKT7-SELENOF′-containing yeast cells and screened by the selection plate for blue colonies; Y2H Gold yeast cells in (E)–(F) were co-transformed with plasmids NpGBKT7-SELENOF′ and preys 15–1(D), 15–2(E), 15–3(F); or with plasmids pGBKT7-Lam and pADT7-T as the negative control (C); or with plasmids pGBKT7-p53 and pADT7-T as the positive control (G), followed by selection on SD/−Trp/−Leu/-His/−Ade/X-α-gal/Aba plates. (DOCX 54 kb) [file 12986_2017_235_MOESM1_ESM.docx]

Additional file 1: Figure S1


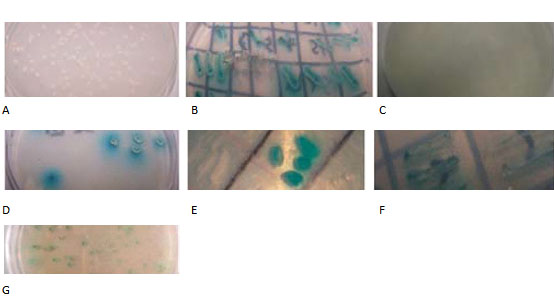


Additional file 1: Figure S1, Yeast two-hybrid screening of the human fetal brain cDNA library using the SELENOF’ gene as a bait. (A) Plasmids carrying the fetal brain cDNA library were co-transformed into the NpGBKT7-SELENOF’-containing yeast cells and screened by the selection plate for blue colonies; Y2H Gold yeast cells in (E)–(F) were co-transformed with plasmids NpGBKT7-SELENOF’ and preys 15-1(D), 15-2(E), 15-3(F) ; or with plasmids pGBKT7-Lam and pADT7-T as the negative control (C); or with plasmids pGBKT7-p53 and pADT7-T as the positive control (G), followed by selection on SD/-Trp/-Leu/-His/-Ade/X-α-gal/Aba plates.
