# Supplementary material for: The interaction of selenoprotein F (SELENOF) with retinol dehydrogenase 11 (RDH11) implied a role of SELENOF in vitamin A metabolism
Source: Nutr Metab (Lond). 2018 Jan 22;15:7. doi: 10.1186/s12986-017-0235-x (PMC5778809; doi:10.1186/s12986-017-0235-x)
Supplement: Supplementary file 2 — Expression level of recombinant SELENOF-HA or RDH11-YFP. The overexpressed SELENOF-HA or RDHA11-YFP was detected by anti-HA antibody (A) or anti-GFP antibody (B). In part A, lane 1 shows HEK293T cells co-transfected with SELENOF-HA-SelExpress1 and RDH11-YFP; lane 2 shows HEK293T cells co-transfected with plasmids SELENOF-HA-SelExpress1 and EYFP empty vector. In part B, lane 1 shows HEK293T cells co-transfected with plasmids SELENOF-HA-SelExpress1 and RDH11-YFP; lane 2 shows HEK293T cells co-transfected with plasmids HA-SelExpress1 empty vector and RDH11-YFP. (DOCX 84 kb) [file 12986_2017_235_MOESM2_ESM.docx]

Additional file 2: Figure S2

A B


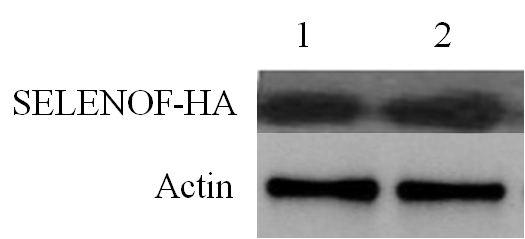

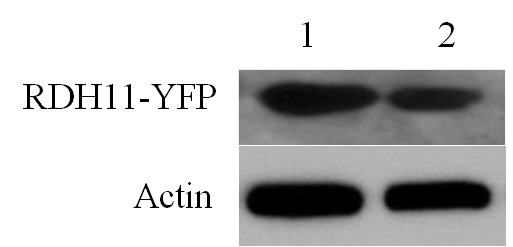


Additional file 2: Figure S2, Expression level of recombinant SELENOF-HA or RDH11-YFP. The overexpressed SELENOF-HA or RDHA11-YFP was detected by anti-HA antibody (A) or anti-GFP antibody (B). In part A, lane 1 shows HEK293T cells co-transfected with SELENOF-HA-SelExpress1 and RDH11-YFP; lane 2 shows HEK293T cells co-transfected with plasmids SELENOF-HA-SelExpress1 and EYFP empty vector. In part B, lane 1 shows HEK293T cells co-transfected with plasmids SELENOF-HA-SelExpress1 and RDH11-YFP; lane 2 shows HEK293T cells co-transfected with plasmids HA-SelExpress1 empty vector and RDH11-YFP.
