# Supplementary material for: The interaction of selenoprotein F (SELENOF) with retinol dehydrogenase 11 (RDH11) implied a role of SELENOF in vitamin A metabolism
Source: Nutr Metab (Lond). 2018 Jan 22;15:7. doi: 10.1186/s12986-017-0235-x (PMC5778809; doi:10.1186/s12986-017-0235-x)
Supplement: Supplementary file 3 — The reductase activity of SELENOF’ toward all-trans-retinaldehyde. The plasmid SELENOF’-PET28b was transfected into E.coli (BL21) and recombinant SelenoF’ was induced and then the cell lysates were used to detect the enzyme activity to reduce all-trans-retinaldehyde (column A); Empty vector PET28b was transfeced into E.coli (BL21) and used as the negative control (column B). (DOCX 53 kb) [file 12986_2017_235_MOESM3_ESM.docx]

Additional file 3: Figure S3


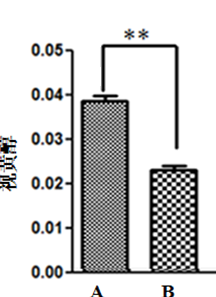


Ratio of retinol

**Additional file 3: Figure S3**, The reductase activity of SELENOF’ toward all-trans-retinaldehyde. The plasmid SELENOF’-PET28b was transfected into *E*.c*oli* (BL21) and recombinant SELENOF’ was induced and then the cell lysates were used to detect the enzyme activity to reduce all-trans-retinaldehyde (column A); Empty vector PET28b was transfeced into *E*.c*oli* (BL21) and used as the negative control (column B).
